# Supplementary material for: Group discussions improve reliability and validity of rated categories based on qualitative data from systematic review
Source: PLoS One. 2025 Jun 18;20(6):e0326166. doi: 10.1371/journal.pone.0326166 (PMC12176165; doi:10.1371/journal.pone.0326166)
Supplement: S1 File — Instructions for raters on objectives of review and data entry. (PDF) [file pone.0326166.s001.pdf]

S1A: Cheat Sheet: Inter-rater-reliability for conservation planning literature: definition of categories to extract

Short concept:
When conservation actions get planned, different types of information and data are used, often within a strategic conceptual planning process. This cheat sheet defines different types of information that are in general important during decision-making and gives options for classifications within the coding table, to be used in the data entry form in the MS-ACCESS-database.

Most important:

1) You only want to extract information that was important during the planning for a distinct case study!
Only information that was directly and explicitly used in some way to come to a decision (for example, as a parameter or input in a model) should get noted. The focus hereby lies on the conservation asset (the thing we care about), and the actions that should be taken for its benefit.
If for example a threat or an objective is in general mentioned as important, or stated that it was used, but is then not part of the description, (= not included in the modeling/decision making process), then it does not count and should not appear in the field. Please note in the comment box if you come across a paper where the authors are not following up with details on their initial statements.

2) Please do not interpret
Only extract what is explicitly stated. Of course, many plans could be used in other context, and you will have many associations come up in your mind when you read a text, but please do only fill in what is explicitly stated in the text for the described case-study.

Technical notes:

MS ACCESS does not perform well with shortcuts (control z/ control/y), please do not use any to avoid frustration. Email me if anything does not work as intended!
Reading vs. skimming: Due to the large amount of literature, strategic reading is recommended: read abstract and method section in detail, use keyword search for all fields that are still empty afterwards. In the case of very long and unstructured papers, skim the whole paper to find the relevant section, which can then be read in detail. Appropriate keyword search terms are suggested in the cheat sheet.

One entry in the database correlates to one decision: If a publication described several examples or case-studies of a decision problem that were not used within a scenario analysis for the same decision, the record has to be duplicated in order to have one data entry per described examples, not one entry per publication. Enter in comment-box, for example: *decision example 2 of 3 within paper xy.* Use the little arrows at the bottom right of the entry form to create a new entry at the end of the table, and do not forget to enter author and title as well for each new entry.
