# Supplementary material for: Group discussions improve reliability and validity of rated categories based on qualitative data from systematic review
Source: PLoS One. 2025 Jun 18;20(6):e0326166. doi: 10.1371/journal.pone.0326166 (PMC12176165; doi:10.1371/journal.pone.0326166)
Supplement: S1 Table — Coding scheme cheat sheet. Full list of classification categories and explanation. (PDF) [file pone.0326166.s002.pdf]

S1B Table: All categories and subcategories to use in the classification of text from manuscripts into an MS ACCESS database, including definitions and examples

Field name	Categories	Definition	Example/potential difficulty	Example entry & keyword search
Type of decision (for consistency check with fields “count objectives” and “count actions”	Single objective, single action<br>Multi objective, single action<br>Single objective, multi action<br>Multi objective, multi action<br>Other reason included	*What is considered in a decision and compared/chosen between or traded off against each other?*<br><br>**Definition of objective:** Objective that is clearly stated in text and describes what the goal of the management is, including a description of how this is tracked / linked to the decision of actions<br><br>**Definition of action:** An action is an option to be evaluated through decision process and be decided between (should I do A or B?). Implementing a set of actions together to achieve a goal does not make it multi action if there is no selection process on which one to do. Only if there is a discrimination between options that have to be decided on makes a decision multi action.	**Objective:** Are multiple objectives given and tracked through the decision process? These can be targets for different species, or other aspects like costs or ecosystem services etc. which will appear for example in a ranking of options that shows clearly how well which option achieves which objective<br><br>**Action:** is there a decision process to compare/decide between options, like building a fence to keep predator out OR baiting to kill a predator. Options can be very similar like building 1 fence or 2 fences, or killing 10% of weed or 40%, or building the fence at location A or B.<br><br>**Difficulty**: If several scenarios are done that have different objectives and/or actions, please use the overall sum	Tickbox
impl_sugg_sci	impl = Implemented	Explicitly stated that this was implemented or is about to get implemented<br>When a case study has been done: if this was tested in small scale and is now recommended to be done at a large scale, this would still count as *scientific applied* if done without any collaboration with people who would be able to implement it, and *suggested* if collaboration existed. *Implementation* means that it is rolled out over the whole area/population of interest	Skim abstract, intro and discussion for any mentioning of collaboration or implementation, check authorlist for departments or institutes outside of academia as a hint, although that is not enough on its own to justify and entry as “impl”	impl<br><br>use keyword search: ”implement”
	sugg = Suggested trial	= tested/experiment at small scale or calculated **in collaboration** with people who have power to make		sugg

		decision about implementation (but more than just a co-author in the department!)		
	scia = scientific applied	= experiment at small scale **without** collaboration with people who have power to make decision		scia
	scit= scientific theoretical	= theoretical science / calculated **without** collaboration with people who have power to make decision		scit
primary framework	SDM = structured decision making<br>SCP = Systematic conservation planning<br>AM = Adaptive management<br>MCDA = Multi-criteria decision analysis<br>CE = Cost-effectiveness analysis	All of these that were explicitly stated to have been used, divided by comma, sorted alphabetically	Use search function to find and check if only mentioned in theory or explicitly stated that used<br><br>**Difficulty:** Uncertainty what the different framework actually does – not important, just go with what they explicitly say they have used and copy paste that line/paragraph into description box.	AM, CE, MCDA, SCP, SDM
Country		Fill in name		If more then one, separate by comma, alphabetically sorted<br>Type "theoretical" if theoretical paper not set in a specific location
Continent<br>Consistency check with field "country"	1 = Europe<br>2 = Americas (US, Canada, Middle and South)<br>3 = Africa and middle East<br>4 = Asia and Russia and India<br>5 = Australia/NZ<br>6 = other (e.g. Pacific Islands, poles, High seas etc)			If country lies somewhere between and you are not sure, use both numbers and state uncertainty in text field (region)
Type management (focus on the asset we want to benefit)	0 = not mentioned<br>1 = spatial prioritization<br>2 = population management<br>3 = threat abatement<br>4 = restoration<br>5 = other	0 = not mentioned<br>1 = spatial prioritization for protection/action (where to do something, or where to have borders of park)<br>2 = population management (provide positive impact from inside of species of concern, e.g. increase numbers/survival, including management of harvest of species)<br>3 = threat abatement (reduce negative impact from human activity/other species outside of species of concern)<br>4 = restoration (focus on habitat/land management, not single species)	**Spatial prioritization PA:**<br>- establishing a PA at priority sites<br>**Species management:**<br>- harvest management of wild mushrooms,<br>- culling buffalo to keep population size within park carrying capacity,<br>- controlling fishing effort<br>- manual pollination of trees,<br>- artificial nesting boxes,<br>- clutch manipulation,<br>- supplementary feeding,<br>- disease/parasite management<br>-species reintroduction<br>- ex situ conservation	Choose all that apply, separate by comma, start with smallest number<br><br>Describe in text field to the right: 1 = xxx, 3 = xxx…

		5 = other (e.g. education, awareness, legislation)	- captive breeding,<br>- artificial propagation,<br>- gene banking<br>**Threat abatement:** any type of site/area management like<br>- putting up fences,<br>- control of poachers<br>- invasive/problematic species control<br>- cutting vines off trees,<br>- preventing pollution<br>- preventing unintentional killing like roadkill<br>**Restoration:** habitat and natural process restoration like<br>- creating forest corridors,<br>- prairie re-creation,<br>- riparian tree plantings,<br>- coral reef restoration,<br>- proscribed burns,<br>- dam removal, fish ladders	
Socio economic objectives	y/n	To choose from opportunity cost, management cost, recreation, ecosystem services, support, or other		
Type **environmental**, **economic**, **social and other** objectives (4 separate fields with the same categories, 1-4 were counted as quantitative)	0 = not clearly stated<br>1 = maximize *(mathematical formula)*<br>2 = minimize *(mathematical formula)*<br>3 = depends on combination with other objective<br>4 = target/threshold<br>5 = other<br>6 = increase	*Environmental objective means here: objective for a natural asset (something that is supposed to benefit from action).*<br><br>Objective for threats-only actions are NOT of interest here! If an action is only targeting a threat without linking it explicitly to a natural asset that should benefit, the environmental objectives field stays empty (0)<br><br>**Minimisation and maximization** are mathematical optimization procedures that require a technical formula. If people just try to somehow increase presence/numbers but measure that not via a rigorous mathematical procedure, classify as 6	Example for 3: area per dollar spent: this is neither maximizing area nor minimizing cost, but the best option depends on the combination<br><br>Example for 5: vague description like increase or benefit without further specification	Choose all that apply, separate by comma, start with smallest number<br><br>Separate lines for, economic, social and environmental objective<br><br>Describe in text field to the right: 1 = xxx, 3 = xxx…<br><br>Keyword*: "max", "min", "target", "thresh", increase"*
Count objectives	1 = 1<br>2 = 2<br>3 = 3 or 4<br>5 = 5 or more	*How many objectives are explicitly stated?*<br><br>*That should relate to checkboxes upper left corner, and to the more detailed boxes on objective types on the lower left side*		Keyword: *"objective"*

alternative actions count	1 = 1<br>2 = 2<br>3 = 3 or 4<br>5 = 5 or more	*That should relate to checkboxes upper left corner*<br><br>Count of options **that are compared to each other as options of choice**: can be single actions, or set of actions that are planned to get implemented together.	A "do nothing" option counts as option, and a decision between doing one action or not doing it is classified as 2.<br>Only papers that plan for one action that do not compare this to a do-nothing-option get classified as 1.	Describe in text field to the right: 1 = xxx, 2 = xxx…, 3 = xxx, 5=xxx
Explicit trade-off attempted between options	Yes/no	*A trade-off is an explicit comparison that attempts to balance between (potentially opposing) objectives: you can't have it all, but you try to find out what option gets you the best mix of all the things you care for. Often different objectives or different opinions have to be combined to come to a conclusion during a tradeoff, which is addressed by information entered in fields below (tradeoff aggregation type and type ranking)*		Search keyword: *tradeoff, trade-off*
Sensitivity or scenario analysis type<br><br>*What is done to test if the result might be different under other circumstances? What is changed in the calculation to test this?*<br><br>Any entry was counted as "yes"	0= no scenario or sensitivity analysis mentioned<br>1 = epistemic uncertainty /measured parameter values changed within model/calculation<br>2 = different models used<br>3 = weights changed within model/calculation<br>4 = scores changed within model/calculation<br>5 = scenarios for different actions (but same calculation) → (likely if you checked multi action)<br>6 = objective set (but same calculation)<br>7 = other<br>8 = spatial inclusions/exclusion (but same calculation)<br>9 = scenarios for different targets (but same calculation)	*The definitions of "scenario analysis" and "sensitivity analysis" are not crystal clear and somewhat overlap. Most important: they describe an attempt of finding out if the result would be different, if you make slight changes during the procedure. This field should contain a description of what has been tested if it results in a change.*	Epistemic uncertainty is uncertainty that results from natural variation: for example testing a model for different age, size or survival of an animal.<br>A different model would mean that for example 2 different softwares, or a math model and expert elicitation have been compared	Choose all that apply, separate by comma, start with smallest number<br><br>Describe in text field to the right: 1 = xxx, 3 = xxx…<br><br>Keyword: *"sensi", "scenario"*
