# Supplementary material for: Group discussions improve reliability and validity of rated categories based on qualitative data from systematic review
Source: PLoS One. 2025 Jun 18;20(6):e0326166. doi: 10.1371/journal.pone.0326166 (PMC12176165; doi:10.1371/journal.pone.0326166)
Supplement: S2 Table — Details on all changes made after the pilot phase to the coding scheme including reasons. (PDF) [file pone.0326166.s003.pdf]

S2 Table: changes after pilot

Changes made after the pilot phase to the coding scheme.

Variable	Pilot categories	Updated categories	Reason
Applied conservation / academic	Implemented<br>Suggested<br>academic	Implemented<br>Suggested<br>Scientific applied<br>Scientific theoretical	Applied academic studies were often categorized as implemented, because academic was linked to theoretical
Spatial scale	State/county level	Scrapped	Too often unclear if locations were truly rolled out over the whole state/county, better to classify as local or regional
Threat		Some explanation for land use change, human use, harvest and system medication added	Confusion about difference of harvest and system change for e.g. water extraction for irrigation, and if this fits to agriculture, harvest or system change
Realm	Terrestrial, marine, freshwater and combinations	Option added for "not mentioned"	To make clearer if entry was forgotten or decided to be NA
Type environmental/ economic/social/other objective	Codes 1 -5	Added:<br>6: increase	Seemed to be too frequent to be "other"

The variable related to implementation was collapsed to "applied conservation" and "academic" and the biodiversity related variable was further collapsed in studies that used the dataset, because of low agreement in the intermediate categories (Beher et al 2024).

References

Beher J, Treml E, Wintle B. 10 years of decision-making for biodiversity conservation actions: A systematic literature review. Conserv Sci Pract. 2024 Jun 22; e13170.
