# Supplementary material for: Group discussions improve reliability and validity of rated categories based on qualitative data from systematic review
Source: PLoS One. 2025 Jun 18;20(6):e0326166. doi: 10.1371/journal.pone.0326166 (PMC12176165; doi:10.1371/journal.pone.0326166)
Supplement: S2 File — (PDF) [file pone.0326166.s004.pdf]

S3. Calculations for figures

Fig 1: Average of 4 pairs: TRUE / FALSE entries between each additional rater compared to main rater for 21 publications for each variable, before and after discussion:

Each rater has the same (TRUE) or different (FALSE) entry in each of the 21 publications for each variable, which is expressed as value between 0 and 21 in column T_F. In a dataframe with columns *rater, variable, before/after, T_F*. Percent agreement is calculated in line 68 in code: fig1_comb$pp <- 100/21 * fig1_comb$T_F

Fig 2: a): all pairwise comparison (each additional rater vs main rater) averaged for each variable. Line 113 in code (sum of all TRUE entries for each rater and each variable, then take average across 5 raters for each variable)

Rare: variables were coded with "FALSE" more than 50% (>52 out of 105)

frequent: variables were coded with "FALSE" less than 20% (< 21 out of 105)

b) all variables averaged for each rater

line 147 in code: (sum of all TRUE entries for each rater and each variable, then take average across all variables for each rater)

Fig 3 - 5: kappa and percent agreement were calculated in R with package *irr* for all combinations of groups between 1-4 additional raters
