# Supplementary figures and images for: Group discussions improve reliability and validity of rated categories based on qualitative data from systematic review
Source: PLoS One. 2025 Jun 18;20(6):e0326166. doi: 10.1371/journal.pone.0326166 (PMC12176165; doi:10.1371/journal.pone.0326166)

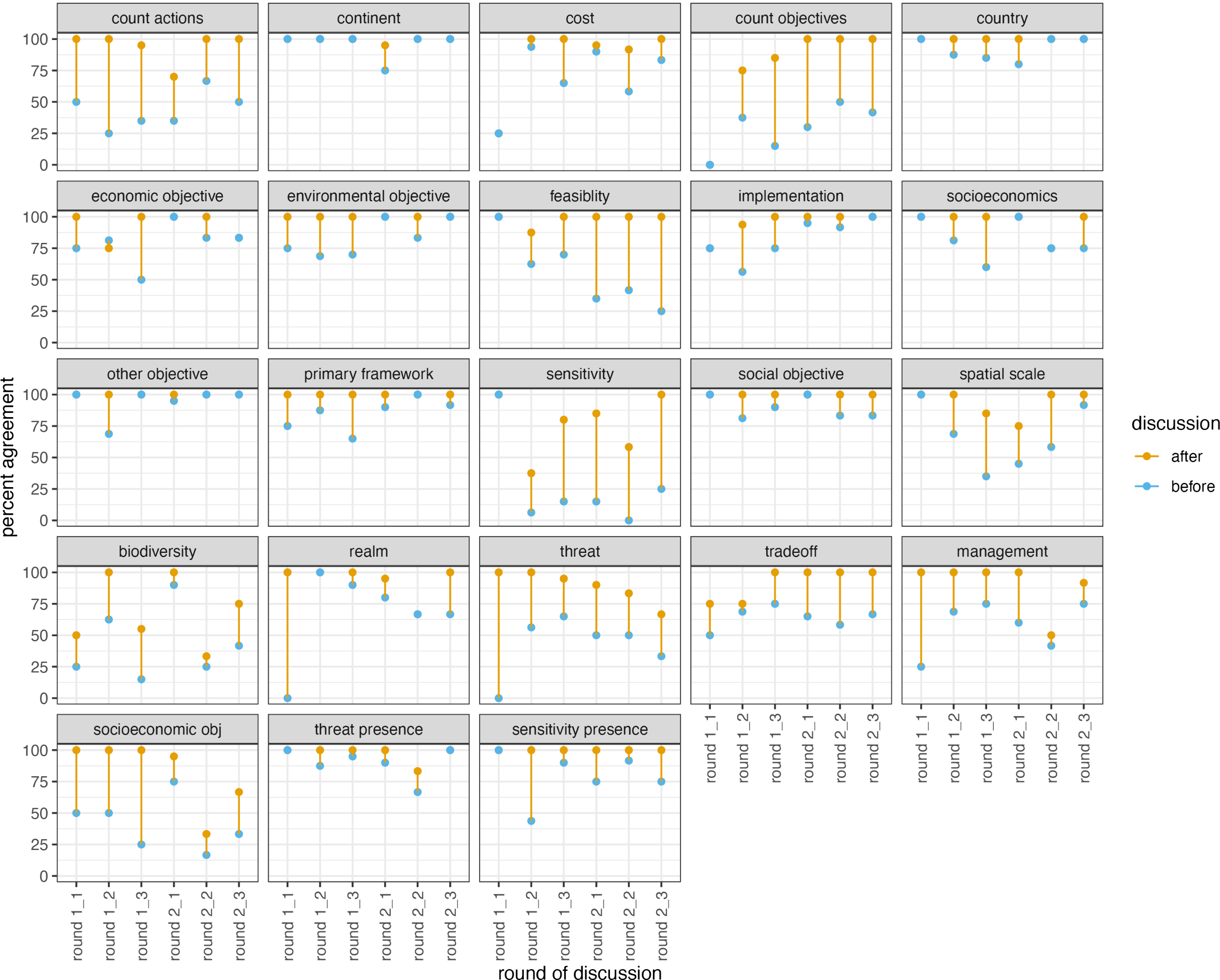

Supplement: S1 Fig — Average agreement across all pairs of additional raters compared to the main rater before and after each of the 6 rounds of group discussions. Discussed examples from publications were n = 1 during the first discussion, and between n = 3–5 in all other discussions. (TIF) [file pone.0326166.s005.tif]
