# Supplementary material for: Group discussions improve reliability and validity of rated categories based on qualitative data from systematic review
Source: PLoS One. 2025 Jun 18;20(6):e0326166. doi: 10.1371/journal.pone.0326166 (PMC12176165; doi:10.1371/journal.pone.0326166)
Supplement: S3 Table — Kappa for individual raters and all raters, alpha for all raters, and percent agreement before and after discussion. The percent agreement after discussion differs drastically for some variables from kappa or alpha. (PDF) [file pone.0326166.s006.pdf]

S5 Table: Comparison of metrics

Metrics for agreement: Kappa for individual raters and all raters, Alpha for all raters, and percent agreement before and after discussion. The percent agreement after discussion differs drastically for some variables from kappa or Alpha.

**variable**	**kappa R1**	**kappa R2**	**kappa R3**	**kappa R4**	**kappa all**	**Alpha all**	**pp before**	**pp after**
count actions	0.2	0.2	0.3	0.1	0.3	0.3	14.3	81.0
continent	0.9	0.8	1.0	1.0	0.9	0.9	85.7	95.2
cost	0.6	0.6	0.5	0.7	0.6	0.6	57.1	85.7
count objectives	-0.1	0.2	0.1	0.1	0.2	0.2	4.8	85.7
country	0.8	0.9	0.9	0.8	0.8	0.8	71.4	100.0
economic objective	0.6	0.5	0.4	0.7	0.5	0.5	57.1	90.5
environmental objective	0.4	0.1	0.7	0.7	0.3	0.3	52.4	100.0
feasibility	0.3	0.1	0.3	0.0	0.2	0.2	14.3	95.2
implementation	0.1	0.6	0.6	0.5	0.4	0.4	57.1	90.5
socioeconomic obj	0.6	0.6	0.3	0.8	0.5	0.5	52.4	95.2
other objective	-0.1	0.0	-0.1	0.0	0.0	0.0	85.7	100.0
primary framework	0.8	0.8	0.8	0.8	0.8	0.8	57.1	100.0
sensitivity presence	0.1	0.1	0.2	0.0	0.1	0.1	4.8	57.1
sensitivity	0.5	0.6	0.8	0.8	0.5	0.5	66.7	100.0
social objective	0.1	0.3	0.2	0.3	0.4	0.4	38.1	71.4
spatial scale	0.3	0.4	0.5	0.5	0.4	0.4	19.0	66.7
biodiversity	0.5	0.8	0.5	0.5	0.6	0.6	57.1	85.7
realm	0.4	0.3	0.4	0.5	0.4	0.4	33.3	81.0
threat	0.4	0.2	0.3	0.3	0.3	0.3	28.6	90.5
threat presence	0.3	0.4	0.8	0.6	0.5	0.5	33.3	85.7
tradeoff	0.2	0.5	0.0	0.4	0.3	0.3	23.8	81.0
management	0.7	0.3	0.9	0.9	0.6	0.6	76.2	95.2
socioeconomic objective	0.2	-0.2	0.3	0.1	0.2	0.2	47.6	100.0
