# Supplementary material for: Group discussions improve reliability and validity of rated categories based on qualitative data from systematic review
Source: PLoS One. 2025 Jun 18;20(6):e0326166. doi: 10.1371/journal.pone.0326166 (PMC12176165; doi:10.1371/journal.pone.0326166)
Supplement: Attachment — Submitted filename: Response to Reviewers.docx [file pone.0326166.s007.docx]

PONE-D-24-38272

Group discussions improve reliability and validity of rated categories based on qualitative data from systematic review

PLOS ONE

Please find in the following a detailed response to all suggestions, referring to line numbers in the manuscripts where changes have been made, and edited text in blue.

**PLOS ONE Journal Requirements:**

When submitting your revision, we need you to address these additional requirements.

1. Please ensure that your manuscript meets PLOS ONE's style requirements, including those for file naming. The PLOS ONE style templates can be found at

<https://journals.plos.org/plosone/s/file?id=wjVg/PLOSOne_formatting_sample_main_body.pdf>

and

<https://journals.plos.org/plosone/s/file?id=ba62/PLOSOne_formatting_sample_title_authors_affiliations.pdf>

I have checked the guidelines for text and figure files and made the following changes:

- Updated formatting of list of authors and affiliations to remove addresses and postcodes and use appropriate symbols for corresponding author and equal set of authors and current addresses
- Updated all level 1 headings to be fontsize 18 (in addition to be bold)
- Changed all references to figures from “Figure” to “Fig” in manuscript and Supplementary material
- Transferred all Annexes from main manuscript to individual files and edited Supplementary material section to provide correct naming convention including titles and captions for figures and tables.
- Changed bolded sentences in discussion paragraph to subheading level 2, and edited some sentences to make it work (line 538: added “This was the case” and removed the comma after first part of sentence that become the subheading in line 536).

While revising your submission, please upload your figure files to the Preflight Analysis and Conversion Engine (PACE) digital diagnostic tool, https://pacev2.apexcovantage.com/. PACE helps ensure that figures meet PLOS requirements. To use PACE, you must first register as a user. Registration is free. Then, login and navigate to the UPLOAD tab, where you will find detailed instructions on how to use the tool. If you encounter any issues or have any questions when using PACE, please email PLOS at figures@plos.org. Please note that Supporting Information files do not need this step.

Thank you for these instructions, I have uploaded all figures and downloaded the improved versions that all passed. I have added the images via the editorial manager.

3. Please ensure that you refer to Figure 1a in your text as, if accepted, production will need this reference to link the reader to the figure.

Following the comment of reviewer 2, the section of text that would have referred to the figure has been deleted

4. Please include a caption for figure 1a.

Please see reply above.

5. Please include a copy of Table 2 which you refer to in your text on page 9.

Please see reply above, the text has been deleted.

6. Please include captions for your Supporting Information files at the end of your manuscript, and update any in-text citations to match accordingly. Please see our Supporting Information guidelines for more information: http://journals.plos.org/plosone/s/supporting-information.

Many thanks for the instructions, I have updated all supplementary material information according to the link and provided headlines and captions in the manuscript as well as individual files for each as uploaded document. I have updated the in-text references accordingly in marked up changes.

7. Please review your reference list to ensure that it is complete and correct. If you have cited papers that have been retracted, please include the rationale for doing so in the manuscript text, or remove these references and replace them with relevant current references. Any changes to the reference list should be mentioned in the rebuttal letter that accompanies your revised manuscript. If you need to cite a retracted article, indicate the article’s retracted status in the References list and also include a citation and full reference for the retraction notice.

I have double checked all references. I noticed that I had one publication in the reference list with 2 numbers (46 & 48, and 7&23) and removed the second entry for each. A few references had some spelling errors and capitalization, which I changed. References for R packages were updated (line 918 (28), and line 922 (30), and one reference added due to new text (Beher et al 2024 in the methods, #25).

Additional Editor Comments :

I've received feedback from two reviewers. Both found your manuscript to be interesting, relevant, and well-written. I agree with them. The reviewers had some comments that you should address, but these are mostly minor and should be straightforward to resolve during revision.

Specific comments:

Line 73: "judgment" instead of "judgement" (for consistency with elsewhere in the text)

Thank you for catching this inconsistency, we have corrected the spelling (now line 74).

Line 231: insert "of" after "consisted"

Thank you for catching this omission, we have added the word (now line 250).

Line 510: replace "couldn't" with "could not"

Thank you for the suggestion, the change was made (now line 533).

[Note: HTML markup is below. Please do not edit.]

Reviewers' comments:

Reviewer's Responses to Questions

Comments to the Author

1. Is the manuscript technically sound, and do the data support the conclusions?

The manuscript must describe a technically sound piece of scientific research with data that supports the conclusions. Experiments must have been conducted rigorously, with appropriate controls, replication, and sample sizes. The conclusions must be drawn appropriately based on the data presented.

Reviewer #1: Yes

Reviewer #2: Yes

________________________________________

2. Has the statistical analysis been performed appropriately and rigorously?

Reviewer #1: I Don't Know

Reviewer #2: Yes

________________________________________

3. Have the authors made all data underlying the findings in their manuscript fully available?

The PLOS Data policy requires authors to make all data underlying the findings described in their manuscript fully available without restriction, with rare exception (please refer to the Data Availability Statement in the manuscript PDF file). The data should be provided as part of the manuscript or its supporting information, or deposited to a public repository. For example, in addition to summary statistics, the data points behind means, medians and variance measures should be available. If there are restrictions on publicly sharing data—e.g. participant privacy or use of data from a third party—those must be specified.

Reviewer #1: Yes

Reviewer #2: Yes

________________________________________

4. Is the manuscript presented in an intelligible fashion and written in standard English?

PLOS ONE does not copyedit accepted manuscripts, so the language in submitted articles must be clear, correct, and unambiguous. Any typographical or grammatical errors should be corrected at revision, so please note any specific errors here.

Reviewer #1: Yes

Reviewer #2: Yes

________________________________________

5. Review Comments to the Author

Please use the space provided to explain your answers to the questions above. You may also include additional comments for the author, including concerns about dual publication, research ethics, or publication ethics. (Please upload your review as an attachment if it exceeds 20,000 characters)

Reviewer #1: This paper presents an interesting investigation into using group discussions to improve the coding of papers in systematic reviews in conservation. I particularly appreciate the fact that the author paid attention to pragmatic costs and feasibility in their analyses which are important considerations. I don't have much to offer in the way of specific improvements.

My one main comment is that in this day and age, the potential for combining human reviewer efforts (including group discussions) with machine-based reviews seems like it is a critical topic that needs to be addressed in this paper. I realize that the actual study being reported in this paper did not use AI tools and I don't think it would be at all feasible to add them into the mix at this point. But I would expect to see at least a couple of sentences in the discussion talking about the potential of combining human and machine based reviews - perhaps a topic for a future study???

Thank you for bringing up this important topic – we agree that since the time of the work, AI has made incredible progress and applications increase in unseen speed and numbers. While we are not experts in the field, we have added a brief paragraph in the discussion section after having spoken with colleagues that work in the field and sighting of recent literature, see line 755ff:

## **The progress of artificial intelligence and machine learning and its potential for automatization of qualitative judgments of texts**

Since this study was conducted, machine learning and artificial intelligence applications that digest text for specific purposes have accelerated in their development and application. While problems like hallucinations persist and high accuracy at scale required for most academic work has not been achieved yet, programs become progressively better in text-related tasks, especially extraction of information from text (54–57). Despite the persisting challenges, researchers see an increasing role of AI in research and for reviews in particular (58). Future studies could compare results across human and electronic raters to assess and monitor if and when these tools will be of the needed quality to apply for contextual classification tasks.

Reviewer #2: This is a really interesting and important paper. It is well written and makes some useful arguments. I am happy to recommend the paper be published if the authors can attend to some minor amendments and suggestions below:

Methods: I agree with the authors on pg 8 lines 178-184 that the information about the systematic review and PRISMA figure can be removed. Instead the authors could provide a short description of the original study and reference it (since it has been published)

We welcome the suggestion to replace the statement about PRISMA with a summary, and have replaced the section with the following text as an introduction to the methods:

Line 220ff:

*To assess the impact of group discussion on the quality of categorical classification of text, we drew on a published dataset of decision-making processes for conservation management (25). The study sampled the peer-reviewed literature with several search strings to find published texts on conservation management decisions that were derived through a prioritization process. The first author coded all texts for several variables and respective categories, requiring some degree of interpretation, and provided quantitative summaries of these categories as results. The group discussions were used as a means to validate the quality of the codes, with the author (in the following referred to as “main rater”) facilitating the experiment and comparing their own ratings and arguments with a group after independent parallel coding.*

Some suggestions:

1. On pg 10, subsection titled Workflow – the authors refer to 25 studies used to illustrate their argument as ‘examples’ – this is a bit confusing – I would suggest they use the terms 25 studies, and coding on 23 categories (were there sub-codes?) and amend all references to ‘examples’ as used above to studies or publications (to make it absolutely clear what they are referring to).

Subsequently the authors refer to ‘case study’ and it is not clear what they are referring to – an ‘example’ or results of a round of coding?

Many thanks for highlighting confusing descriptions. We have changed the text throughout accordingly.

2. Perhaps the author/s could mention who the main rater was – and their expertise that made them the main rater earlier in the paper (it comes very late in the discussion). It would also be useful to mention whether any training was given to the additional raters before the process and whether they all had domain expertise (albeit to varying levels).Although on pg 27 it is mentioned that in case the studies being coded are very heterogeneous, training of coders can be difficult so as to improve coding agreement – nevertheless some initial training needs to have been provided and some mention of that would be useful.

Many thanks for these suggestions to describe the process more clearly.

We have included some text in the new introduction to the methods to explain the role of the main rater in line 220 ff, as described in response to the reviewer comment in line 166 in this document.

Reference to training is also given in line 245 (we added “*To provide some introduction and training*,” to make it more clear that the pilot study aimed at providing training), we also provided information on the expertise of the students in line 238 (“*All raters were students of environmental sciences with a focus on conservation*.”) and pulled up text from line 262ff to line 239ff have all information regarding expertise and training of the group in one paragraph.

3. Pg 10: line 245 – perhaps the authors would like to clarify whether each time different 3 -5 studies were used in each round of subsequent coding – [it is a little confusing whether all studies were coded only once by coders (in different rounds) or some studies were coded twice or more in different rounds.

Thank you for pointing out that the text is not clear enough. We have added the following and hope this describes the process in more clarity:

Line 252ff: *The work was split up into several sessions with a duration of a few hours each to allow raters to remember the details of the rated publications for the group discussion, before rating the next set of publications. Six rounds of coding were needed to work through all publications , each consisting of individual rating of text followed by a group discussion.*

4. From the description it appears to me that the group discussion took place only after all the rounds of coding were completed – If that was the case, I am not sure what was the point of doing the coding piecemeal in rounds? It is a bit unclear.

Please see the response to the previous point, we hope the edits have made it clearer that the consecutive rounds enabled raters to remember the studies and have sessions that were not too long.

5. Were the authors able to conclude whether there is an optimum number of additional coders – (marginal benefit of additional coders and at what point does it become negative)? It might also be worth speculating whether the marginal cost of an additional coder (time, effort and funds) is worth the marginal benefit?

That is a very good question and would be likely to be easier to answer if more studies would exist that test for the agreement rates between different additional raters. Very likely, individual characteristics of each person matter, such as prolonged concentration span and attention to detail, but in some cases also knowledge of specific terminology and how it relates to the classification codes at hand. For example, if only one student can be hired as a comparison, it might come down to bad luck if this student might be a poor performer, but with a larger group, it becomes more likely that there is a range of skill and dedication present in the group. As can be seen in figures 4 and 5, having only one additional rater likely will not catch as many possible disagreements as 2,3 or more additional raters. I see this as you suggest as well, as a trade-off between feasibility and certainty. As the study shows, a test with additional raters can lead to the insight that some categories should be rather collapsed as there are high disagreement rates among independent raters. In the case of this study, the produced codes of the main author passed the test of scrutiny, however, I would not assume that is necessarily always the case. I have added some text in line 751ff:

*In light of our results, we highly recommend including a targeted inter-rater-reliability test in any classification task from text that goes beyond kappa statistics. Some issues of classification are not related to specific coder experience or skill but are inherent in free-flowing text documents, including unclear structure and terminology. Having more than one rater to compare error rates against will improve understanding of the quality of code and improve trust in quantitative and qualitative results that stem from sampling of large numbers of texts. As different types of reviews and summaries of literature are specifically part of doctoral studies, we encourage students to seek out existing additional funding sources, and Universities for providing support, to enable a systematic endorsement and best practice of conducting reviews by elevating such studies from solo enterprise to a quality checked group effort.*

6. Pg 25: if coding some categories was more difficult for some coders than others – what does it say about the level of domain expertise additional coders must have. Perhaps the authors would like to make some recommendations?

Thank you for this suggestion. However, as we discuss in on page 25, it was not really indicative of a general issue with lack of knowledge on the side of the additional raters, but more a mix of issues, that were either caused by oversight (often caused by many different cues being scattered throughout a longer text), or ambiguity of the text (inconsistent language or lack of specificity that allows for different plausible interpretations). Neither are linked to expertise or could be solved by hiring a person with a specific skillset. The only recommendation that comes out of the study is that additional raters can bring problematic categories to the attention of the main author of the study, and allow for a better assessment of the uncertainty (as a measure of quality) of the produced codes.

Please also see the added text in line 751ff we refer to in the pervious comment that we added to the conclusion to have a clearer statement as a take home message, including expertise.

7. Finally, perhaps the authors would like to comment on how might PhD candidates who are conducting a systematic review, as part of their thesis, incorporate (and pay for) additional coders and what impact that might have on whether this would be acceptable. Furthermore, would the implications of this study suggest that systematic reviews should not be a solo enterprise?

Thank you for this suggestion. As the work has been part of a PhD project, we agree it might be beneficial to make specific recommendations. Please see the responses to the previous 2 questions and the general take home message in line 751ff.

Just a minor edit: Pg 8 line 173 – Perhaps the authors would like to remove what seems like an accidental reference to the fact that the paper has directly been lifted from a larger thesis.

Thank you for pointing out that oversight, much appreciated. We have replaced the text in the parenthesis with the actual reference of the publication.

________________________________________

6. PLOS authors have the option to publish the peer review history of their article (what does this mean?). If published, this will include your full peer review and any attached files.

If you choose “no”, your identity will remain anonymous but your review may still be made public.

Do you want your identity to be public for this peer review? For information about this choice, including consent withdrawal, please see our Privacy Policy.

Reviewer #1: No

Reviewer #2: Yes: Jyoti Belur

________________________________________

[NOTE: If reviewer comments were submitted as an attachment file, they will be attached to this email and accessible via the submission site. Please log into your account, locate the manuscript record, and check for the action link "View Attachments". If this link does not appear, there are no attachment files.]

I have checked and not seen any link with this name, so I assume the comments in the email where all comments to address. Please let me know if this is not accurate and I overlooked anything.
