# Supplementary material for: Group discussions improve reliability and validity of rated categories based on qualitative data from systematic review
Source: PLoS One. 2025 Jun 18;20(6):e0326166. doi: 10.1371/journal.pone.0326166 (PMC12176165; doi:10.1371/journal.pone.0326166)
Supplement: Attachment — Submitted filename: Response_to_Reviewers_auresp_2.docx [file pone.0326166.s008.docx]

Dear Dr. Koch,

Please find the list of suggested edits from the last review and our responses, in blue, including line numbers, below.

I hope the manuscript meets now the standard of PLOS ONE and can be accepted for publication. If there is anything else, I will be of course more than happy to address any new or other issues.

Kind regards,

Jutta Beher

Additional Editor Comments:

Line 43: I suggest "meta-analyses" instead of the singular "meta-analysis".

Thank you for the suggestion, the change is made.

Lines 65-67: The end of the sentence reads awkwardly. I suggest the following rewrite: "...that we found in our Web of Science search reported completion of any reliability checks or even stated the general importance of doing so."

Thank you for the suggestion, the change is made.

Line 72: Insert "that" between "coding" and "requires".

Thank you for the suggestion, the edit is made.

Line 78: Rewrite: "...interpreting the context in which a word is used and making..."

Thank you for the suggestion, the edit is made.

Line 96: Delete second appearance of "against" on this line.

Thank you for the suggestion, the edit is made

Line 98: Insert "the" between "in" and "form".

Thank you for the suggestion, the edit is made.

Lines 106-108: I suggest moving "therefore" to the beginning of the sentence, i.e., "Therefore, a thorough, rigorous process..."

Thank you for the suggestion, the edit is made.

Line 164: I suggest inserting "by the raters" between "reflection" and "on".

Thank you for the suggestion, the edit is made.

Line 165: I suspect you meant "beliefs" instead of "believes".

Thank you for the correction, the edit is made.

Line 257 and elsewhere: When you mention kappa in the results, are you referring to Fleiss' kappa, Light's kappa, or something else (i.e., an agreement measure that allows for more than two raters)? Please clarify.

Thank you for raising the unclear referencing to the metric. I have edited the sentence in line 257 to: *Fleiss’ kappa (in the following referred to as “kappa”),*

To clarify that here and in the following we refer to Fleiss’ kappa whenever kappa is mentioned.

Line 259: Replace "does" with "do".

Thank you for the suggestion, the edit is now in line 260

Line 261: "Fleiss' kappa" instead of "Fleiss's kappa".

Thank you for the correction, the edit can be seen in line 262.

Line 261-262: "Krippendorff's alpha".

Thank you for the correction, the edit can be seen in line 262.

Line 262: "Scott's pi".

Thank you for the correction, the edit can be seen in line 262.

Line 282: replace semi-colon with a colon.

Thank you for the suggestion, the edit can be seen in line 289.

Line 329: Which kappa?

Please see our edit regarding your comment further up, where we indicate that we refer to Fleiss’ kappa throughout. Please let us know if you prefer to insert “Fleiss’” in front of any mention of kappa instead.

Line 329: "Krippendorff's".

Thank you for the correction, the edit can be seen in line 330.

Line 330: Replace "general" with "generally".

Thank you for the suggestion, the edit can be seen in line 331.

Line 331: Delete "in general".

Thank you for the suggestion, the edit can be seen in line 332.

Line 339: Replace "show" with "showed".

Thank you for the correction, the edit can be seen in line 341.

Line 341: Replace "pass" with "passed".

Thank you for the correction, the edit can be seen in line 342.

Lines 346-347: Which kappa?

Line 373, 378, and 382: Which kappa?

Line 378: Which kappa?

Line 433: Which kappa?

Please see our edit regarding your comment further up, where we indicate that we refer to Fleiss’ kappa throughout. Please let us know if you prefer to insert “Fleiss’” in front of any mention of kappa instead.

Line 434: Replace "doesn't" with "does not".

Thank you for the suggestion, the edit can be seen in line 435.

Line 435: Replace "won't" with "will not".

Thank you for the suggestion, the edit can be seen in line 436.

Line 438: Replace "allows" with "makes it possible".

Thank you for the suggestion, the edit can be seen in line 439.

Lines 442-443: Which kappa?

Please see our edit regarding your comment further up, where we indicate that we refer to Fleiss’ kappa throughout. Please let us know if you prefer to insert “Fleiss’” in front of any mention of kappa instead.

Lines 453-454: Rewrite as "disagreement due to ambiguity".

Thank you for the suggestion, the edit can be seen in line 455.

Line 575: Suggest replacing "used" with "posed".

Thank you for the suggestion, the edit can be seen in line 577.

Line 586: Suggest inserting "careful" or "close" after "pay".

Thank you for the suggestion, the edit can be seen in line 588.

Line 587: Delete "to look out for".

Thank you for the suggestion, the edit can be seen in line 589.

Line 685 ("hallucinations"): I'm not sure what you mean here. What is a hallucination with respect to an AI algorithm / model?

Thank you for the question. “Hallucinations” is a term that describes any errors made by generative AI. This term is frequently used in AI related publications, some chose more blunt terms, including “bullshit”. The term “hallucination” is also used (and described) in several of the citations I refer to. I have added brief explainer in line 687, please let me know if that is not sufficient:

*However, generative AI and chatbots make mistakes, too, and can for example suggest citations that do not exist or write code that does not work. Such errors are often referred to as “hallucinations”.*
